# Molecular characterization of peste des petits ruminants virus in small ruminants from the Savannah District, Northern Ivory Coast

**DOI:** 10.5455/javar.2026.m1041

**Published:** 2026-06-22

**Authors:** Konan Ange-Sylvestre Goli, Aristide Anicet Zobo, Augustino Alfred Chengula, Melvyn Quan, Benjamin Kiffopan M’Bari, Mariam Makange, Jean Nepomuscene Hakizimana, Peter Thompson, Gerald Misinzo

**Affiliations:** 1 Department of Microbiology, Parasitology, and Biotechnology, College of Veterinary Medicine and Biomedical Sciences, Sokoine University of Agriculture, Morogoro P.O. Box 3019, Morogoro 67125, Tanzania; 2 Department of Veterinary Tropical Diseases, Faculty of Veterinary Science, University of Pretoria, Private Bag X04, Onderstepoort 0110, South Africa; 3 SACIDS Africa Centre of Excellence for Infectious Diseases, SACIDS Foundation for One Health, Sokoine University of Agriculture, P.O. Box 3297, Morogoro 67125, Tanzania; 4 National Laboratory for Agricultural Development Support, Bingerville BP 206, Ivory Coast; 5 Animal Biology, Agropastoral Management Institute, Peleforo Gon Coulibaly University, BP 1328, Korhogo, Ivory Coast; 6 OR Tambo Africa Research Chair for Viral Epidemics, SACIDS Foundation for One Health, Sokoine University of Agriculture, P.O. Box 3297, Morogoro 67125, Tanzania; 7 Department of Production Animal Studies, Faculty of Veterinary Science, University of Pretoria, Private Bag X04, Onderstepoort 0110, South Africa

**Keywords:** Ivory Coast, goat, lineage IV, molecular epidemiology, PPRV, sheep

## Abstract

**Objectives:** Peste des petits ruminants (PPR) is a highly contagious viral disease affecting sheep and goats, with significant socio-economic consequences. The disease is endemic in the Ivory Coast; molecular data on circulating strains, particularly in the northern Savannah district, remain limited. This study aimed to characterize the PPR virus (PPRV) strains circulating among sheep and goats in this area.

**Materials and Methods:** A total of 355 nasal swabs were collected from unvaccinated small ruminants, regardless of clinical status, across three regions of the Savannah district (Poro, Tchologo, and Bagoué). Viral ribonucleic acid (RNA) was extracted and screened using real-time reverse transcription polymerase chain reaction (RT-qPCR) targeting the nucleoprotein (*N*) gene of PPRV, followed by conventional RT-PCR (RT-cPCR). Positive amplicons were sequenced using the Sanger method, and phylogenetic analysis was performed with PhyML and edited in MEGA X software.

**Results:** Out of 355 samples analyzed, 25 (7%) tested positive by RT-qPCR, and 17 of these were amplified by RT-cPCR for nucleotide sequencing. Ten sequences were of sufficient quality for phylogenetic analysis. All sequences described in this study clustered within lineage IV of PPRV and shared high nucleotide identity (96-98%) with previously described West African PPRV strains. Phylogenetic reconstruction revealed two distinct sub-clusters, suggesting possible multiple viral introductions, likely facilitated by uncontrolled transboundary livestock movements.

**Conclusions:** This study provides the first molecular evidence of PPRV circulation in the Savannah district, confirming the dominance of lineage IV. The findings underscore the need for enhanced molecular surveillance and cross-border collaboration to inform regional vaccination and eradication efforts.

## 1. Introduction

Peste des petits ruminants (PPR) is one of the most devastating and highly contagious transboundary viral diseases affecting sheep and goats, particularly in developing countries where small ruminants play a crucial role in food security, income generation, and the livelihoods of rural communities [1, 2, 3]. The disease is caused by the PPR virus (PPRV) (*Morbillivirus caprinae*) [4], a member of the *Morbillivirus* genus within the *Paramyxoviridae* family, closely related to the eradicated rinderpest virus (RPV) [2, 5].

Clinically, PPR is characterized by high fever, ocular and nasal discharges, stomatitis, pneumonia, and severe diarrhea, often leading to morbidity rates approaching 100% and mortality rates ranging from 70% to 90% in immunologically naïve populations [1, 2, 5, 6]. The virus spreads mainly through direct contact between infected and susceptible animals via secretions and excretions but can also be transmitted indirectly through contaminated materials and fomites [2, 6, 7].

Peste des petits ruminants has a significant socio-economic impact across Africa, the Middle East, and Asia, where millions of households depend on small ruminant farming for their livelihoods. The disease significantly reduces livestock productivity and trade, prompting the Food and Agriculture Organization of the United Nations (FAO) and the World Organisation for Animal Health (WOAH) to launch a global eradication strategy, targeting the elimination of PPR by 2030 [8, 9, 10]. Strengthening molecular epidemiological knowledge of PPRV remains a central pillar of this strategy, as it provides essential insights for surveillance, vaccine selection, and disease control in endemic regions [9, 11, 12].

Molecular characterization studies have classified PPRV into four distinct genetic lineages (I-IV) [2, 13]. Lineage IV is currently predominant in Asia, the Middle East, and parts of Europe, while lineages I, II, and III have historically circulated in West, Central, and East Africa [14, 15, 16]. However, recent studies highlight increasing transboundary virus movement and shifts in lineage distribution, underscoring the urgent need for continuous molecular surveillance to monitor viral evolution and inform control strategies [16, 17, 18].

In Ivory Coast, the small ruminant population is estimated at over six million heads, with goats accounting for more than four million [19]. The first confirmed case of PPRV in the country dates back to 1942 [20]. Subsequent serological studies have confirmed its persistence, especially in the northern region (e.g., Korhogo), where a seroprevalence of 36.26% was reported in goats in 2020 [21]. Despite this, molecular data on circulating viral strains remain scarce, limiting understanding of the virus’s epidemiology and evolution in the country.

To date, only two complete PPRV sequences from the Ivory Coast have been reported, one belonging to lineage I (1989, Accession number EU267273) [22] and another to lineage II (2009, Accession number KR781451) [23]. In addition, 17 partial nucleoprotein (*N*) gene sequences have been published, including three from lineage II and 14 from lineage IV, obtained from samples collected between 2013 and 2021 across various regions, such as Bouaké, Bocanda, Dimbokro, Bongouanou, Biankouma, Agboville, and Grand-Yapo [15].

The Savannah district represents a major entry point for live animal movements into the Ivory Coast, with more than three million animals imported in 2023 from neighboring countries such as Burkina Faso, Mali, and Niger, where PPR remains highly endemic [24, 25]. Together with the western and central regions, the Savannah district forms the backbone of national livestock production, accounting for approximately 40% of the national herd [26, 27].

Although the Ivory Coast has adopted the FAO-WOAH global PPR eradication strategy, vaccination coverage in the northern regions has remained low. National vaccination campaigns conducted between 2014 and 2019 achieved an estimated coverage of approximately 5% of the small ruminant population, which is insufficient to interrupt viral transmission [28]. In parallel, national dependence on small ruminant imports is estimated at 34%, representing nearly 10,000 metric tonnes annually, further increasing the risk of virus introduction and spread [28].

Moreover, uncontrolled cross-border livestock movements significantly increase the risk of PPRV introduction and spread. In West Africa, the free movement of people and goods within the Economic Community of West African States (ECOWAS) facilitates extensive animal mobility. Although the International Transhumance Certificate provides a regulatory framework, its enforcement is inconsistent [29, 30, 31]. Consequently, herds from Sahelian countries regularly cross into Ivorian territory, particularly through the Savannah district, during the dry season in search of pasture and water.

In this context, the present study aims to characterize the PPRV strains circulating among sheep and goats in the Savannah district of the northern Ivory Coast. The findings are expected to strengthen national molecular surveillance, enhance understanding of viral transmission dynamics in this strategically located cross-border region, and contribute to regional and global efforts aimed at eradicating PPR.

## 2. Materials and Methods

### 2.1. Ethical approval and compliance

Samples used in this study were collected as part of epidemiological surveillance activities coordinated by the National Laboratory for Support to Agricultural Development (LANADA). Prior authorization (N:/1090/MIRAH/DSV/SDSA) was obtained from the Regional and Departmental Directorates of the Ministry of Animal and Aquatic Resources (MIRAH) and the Department of Veterinary Services (DSV).

Although local regulations do not require ethical clearance for livestock epidemiological surveillance, formal approval was granted by competent authorities. Sampling was performed by licensed veterinarians during routine prevention and diagnostic campaigns, with the informed consent obtained from livestock owners. The study’s objectives were clearly explained to each farmer, who voluntarily agreed to participate. No samples were collected beyond those required for standard diagnostic and surveillance purposes.

### 2.2. Study area

**Figure 1.**
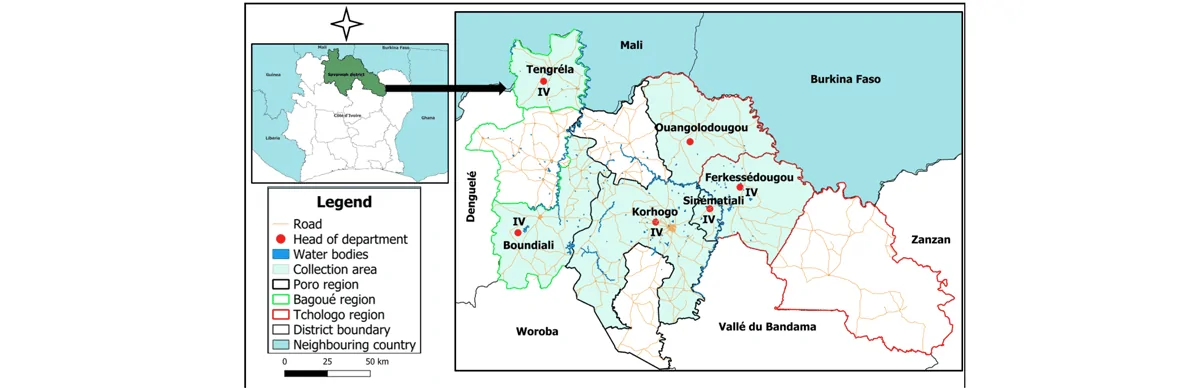
Map showing the Savannah district located in the northern Ivory Coast. The area in green represents the various departments surveyed. The area outlined in red corresponds to the Tchologo region, black to the Poro region, and green to the Bagoué region. The map was generated using QGIS version 3.38, based on OCHA-HDX datasets (version 1.83.6).

This study was conducted in the Savannah district, located in the northern Ivory Coast, covering three administrative districts: Poro, Bagoué, and Tchologo (Figure 1). This district was selected because it represents major livestock-producing areas and is considered at high risk for PPR transmission due to intensive animal movement. The Savannah district is characterized by a Sudano-Sahelian climate, with distinct dry and rainy seasons that influence livestock production and mobility. Livestock farming is widely practiced, with small ruminants (sheep and goats) commonly reared alongside cattle, domestic pigs, and poultry. Sampling sites included livestock markets, villages, and herd aggregation points within the three regions. The district borders Burkina Faso and Mali, and cross-border animal movements occur regularly through both official and informal transhumance routes. These movements were considered when selecting sampling locations, as they may facilitate the introduction and circulation of PPRV. The study area was therefore chosen to capture virus circulation in zones with frequent animal contact and movement.

### 2.3. Sample size determination

The sample size was calculated for a concurrent cross-sectional seroprevalence study using the formula proposed by Ancelle [32]:


1 \[
n\; = \;\frac{{P\left( {1 - P} \right){Z^2}}}{{{i^2}}}
\]

Where, *P* is the estimated prevalence, *Z* is the critical value for a given confidence level, and *i* is the margin of error. Based on a PPR prevalence of 36.26% [21], a 95% confidence level (*Z* = 1.96), and an error margin of 5%, the estimated sample size was 355 small ruminants.

### 2.4. Study design

A cross-sectional survey was conducted from September to December 2023, using a five-stage sampling design. The study targeted areas with high levels of small-ruminant market activity and significant animal imports. In each of the three regions, two departments were randomly selected. Within each department, three sub-prefectures were selected, and three villages were then selected by simple random sampling from the official lists provided by the local veterinary authorities. In each village, 16 unvaccinated small ruminants, as declared by the veterinary officer and/or the owner, were sampled. From each farm, four animals (of either sex, aged at least six months, with age determined by dental examination) were selected regardless of their clinical condition. Nasal swabs were collected following a brief clinical examination.

### 2.5. Sample collection

Nasal swab collection followed the WOAH protocol [3]. Swabs were obtained from live goats and sheep, regardless of the presence of clinical signs suggestive of PPR such as fever, diarrhea, lacrimation, conjunctivitis, or nasal/ocular discharge.

Cotton-tipped swabs mounted on flexible rods were moistened with viral transport media (VTM), inserted into the animals’ nasal cavity, and gently rotated against the mucosa for approximately one minute. Swabs were immediately placed into sterile tubes containing 2 ml of VTM, properly labeled, and kept cool at 4°C on dry ice during transport to the LANADA Regional Laboratory of Korhogo (LRK).

Samples were subsequently stored at –80°C until they were transferred to the Central Veterinary Laboratory of Bingerville (LCVB/LANADA) for molecular analysis.

In the laboratory, samples were vortexed and centrifuged at 5,000 revolutions per minute (rpm) for 10 min at 4°C. The resulting supernatants were collected, labeled according to their original sample information, and stored at –80°C until ribonucleic acid (RNA) extraction.

### 2.6. Viral ribonucleic acid extraction

Total RNA was extracted directly from the VTM containing nasal swab samples using the QIAGEN RNeasy Mini Kit (QIAGEN, Hilden, Germany; Cat. No. 74106) following the manufacturer’s instructions. Extracted RNA was eluted and stored at –80°C until further analysis. A previously confirmed PPRV-positive RNA sample from LCVB/LANADA (Ivory Coast) served as a positive control, while RNase-free water was used as a no-template negative control.

### 2.7. Real-time reverse transcription-polymerase chain reaction (RT-qPCR)

Real-time reverse transcription-polymerase chain reaction (RT-qPCR) was used in this study as a screening assay to detect PPRV RNA. 5 µl of RNA template was amplified using a one-step real-time RT-PCR kit (QIAGEN, Hilden, Germany, Cat. No. 210212) targeting a 351 base pair (bp) fragment of the *N* gene, following the protocol previously described by Kwiatek et al. [33].

The RT-qPCR reactions were performed in a final volume of 25 µl, consisting of 5 µl of RNA template and 20 µl of reaction mixture. The reaction mixture contained 12.5 µl of 2x master mix, 1 µl of reverse transcription enzyme mix, 1 µl each of forward and reverse primers (PPR-NP3 and PPR-NP4; final concentration 10 µM each), 0.5 µl of the NPPR-pb probe (final concentration 0.2 µM), and 4 µl of RNase-free water. The primer sequences were PPR-NP3 (5ʹ–GAG TCT AGT CAA AAC CCT CGT GAG–3ʹ) and PPR-NP4 (5ʹ–TCT CCC TCC TCC TGG TCC TC–3ʹ), and the probe sequence was FAM–5ʹ–CGG CTG AGG CAC TCT TCA GGC TGC–3ʹ–BHQ1 [33]. The amplification was carried out under the following thermal conditions: an initial denaturation at 95°C for 10 min, followed by 40 cycles of denaturation at 95°C for 15 sec, annealing at 60°C for 30 sec, and extension at 72°C for 30 sec. Samples with a cycle threshold (Ct) value ≤ 35 were considered positive and selected for further confirmation and amplification by conventional RT-PCR (RT-cPCR).

Before shipment, 230 µl of RLT Plus buffer was added to 70 µl of purified RNA to stabilize and preserve RNA integrity during transport. Aliquots were sent to the International Atomic Energy Agency (IAEA) in Vienna, Austria, and to Sokoine University of Agriculture (SUA; Morogoro, Tanzania).

### 2.8. Conventional RT-PCR (RT-cPCR)

Upon receipt, samples were subjected to RT-qPCR using the method previously described by Batten et al. [34] to confirm the initial screening results. Samples confirmed as positive were subsequently subjected to RT-cPCR to amplify a 687 bp fragment of the *N* gene of PPRV, suitable for sequencing and phylogenetic analysis, following the protocol described by He et al. [35]. The RT-cPCR was performed using primers PPRNF (5ʹ–ATT GTC CAC TAT TGA ATC CTT GAT–3ʹ) and PPRNR (5ʹ–TTG TCG TTG TAG ACC TGA CTG TTG–3ʹ), as previously described by He et al. [35], each at a final concentration of 0.4 µM. Thermal cycling conditions for RT-cPCR were as follows: reverse transcription at 48°C for 20 min, initial denaturation at 95°C for 10 min, followed by 35 cycles of denaturation at 95°C for 15 sec, annealing at 55°C for 30 sec, and extension at 68°C for 1 min, with a final extension step at 68°C for 5 min. Amplified products were visualized by electrophoresis on a 1.5% agarose gel stained with Safe View dye and documented using a gel documentation system.

### 2.9. Sequencing

The RT-cPCR-positive amplicons were purified and submitted for nucleotide sequencing to Macrogen (Amsterdam, The Netherlands). Sequencing was performed using the Sanger method. Briefly, purified PCR products were processed using a commercial purification kit and sequenced with the BigDye™ Terminator v3.1 Cycle Sequencing Kit (Applied Biosystems). Capillary electrophoresis was carried out on an ABI 3500 Genetic Analyzer (Applied Biosystems, USA).

### 2.10. Sequence processing and phylogenetic analysis

Raw sequence chromatograms were assembled and edited using Pregap4 (v1.6-r) and BioEdit (v7.2.5). Multiple sequence alignments were performed using MAFFT (v7.475), comparing the sequences generated in this study with the corresponding *N* gene region of the complete PPRV reference genome (GenBank accession number NC_006383) and additional reference sequences retrieved from GenBank.

Phylogenetic analysis was subsequently conducted using the Maximum Likelihood (ML) method implemented in PhyML (v3.0) (http://atgc.lirmm.fr/phyml), with node support assessed using the approximate likelihood-ratio test (aLRT) [36, 37, 38, 39]. The resulting phylogenetic tree was visualized, edited, and annotated using MEGA X (V10.2.6). All sequences generated in this study have been deposited in GenBank under accession numbers PX445909–PX445918.

## 3. Results

### 3.1. Detection of peste des petits ruminants virus by RT-qPCR and RT-cPCR

**Figure 2.**
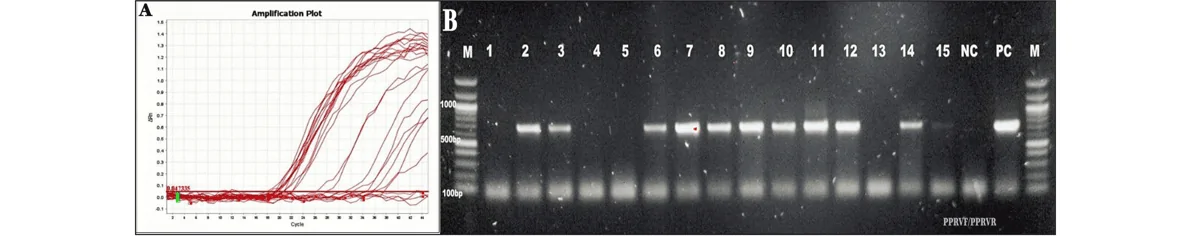
Real-time reverse transcription-polymerase chain reaction (RT-qPCR) fluorescence amplification curves (A) and gel electrophoresis of the conventional RT-PCR (B). The expected amplicon size of the gel was 687 bp. M: 100 bp molecular weight marker; Lanes 1 to 15: test samples; NC: negative control; PC: positive control.

Table 1 presents the characteristics of the 25 samples that tested positive for PPRV by RT-qPCR (Figure 2A), among which 17 tested positive by RT-cPCR (Figure 2B). Of the 17 RT-cPCR positive samples, 10 yielded high-quality nucleotide sequences. These positive samples were collected across the three regions of the Savannah district: 10/25 (40%) in Poro, 8/25 (32%) in Bagoué, and 7/25 (28%) in Tchologo. Regarding host species, goats showed a slightly higher proportion of infections (52%) compared to sheep (48%). The West African Dwarf (WAD) breed accounted for 92% of the cases. Only two positive sheep, both from the Tchologo region, were identified as belonging to the Sahelian breed. In terms of sex distribution, females represented 72% of the positive animals, while males accounted for 28%. Concerning age, animals over two years old accounted for 56% of infected animals, compared to 24% and 20% for animals under two years old and one year old, respectively (Table 1).

**Table 1.** Description of the samples that tested positive for peste des petits ruminants virus by RT-qPCR and conventional RT-PCR (RT-cPCR) in the Savannah district based on the location, species, breed, age, and sex.

Location	Specie	Breed	Age (years)	Sex	RT-qPCR	RT-cPCR	Succesful sequencing	Exploitable sequence	Accession number
Tchologo region									
Ferkessédougou	Sheep	Sahelian	> 2	M	**+**	–	–	–	–
	Goat	WAD	> 2	F	**+**	**+**	Yes	Yes	PX445914
	Goat	WAD	< 2	F	**+**	**+**	Yes	Yes	PX445915
	Sheep	Sahelian	> 2	M	**+**	–	–	–	–
	Sheep	WAD	> 2	M	**+**	–	–	–	–
Ouangolodougou	Sheep	WAD	> 2	F	**+**	–	–	–	–
	Sheep	WAD	> 2	F	**+**	**+**	Yes	No	**–**
Poro region									
Korhogo	Sheep	WAD	< 2	M	**+**	**+**	Yes	Yes	PX445912
	Sheep	WAD	< 2	F	**+**	–	–	–	–
	Sheep	WAD	> 2	M	**+**	**+**	Yes	No	**–**
	Goat	WAD	< 1	M	**+**	–	–	–	–
Sinméatiali	Goat	WAD	< 2	F	**+**	–	–	–	–
	Goat	WAD	< 1	F	**+**	**+**	Yes	Yes	PX445909
	Goat	WAD	< 1	F	**+**	**+**	Yes	No	**–**
	Goat	WAD	< 1	F	**+**	**+**	Yes	No	**–**
	Goat	WAD	> 2	F	**+**	**+**	Yes	Yes	PX445910
	Goat	WAD	< 2	F	**+**	**+**	Yes	Yes	PX445911
Bagoué region									
Boundiali	Goat	WAD	< 2	M	**+**	**+**	Yes	Yes	PX445913
	Sheep	WAD	> 2	F	**+**	–	–	–	–
	Sheep	WAD	< 1	F	**+**	**+**	Yes	Yes	PX445916
	Sheep	WAD	> 2	F	**+**	**+**	Yes	No	**–**
Tengréla	Goat	WAD	> 2	F	**+**	**+**	Yes	No	**–**
	Goat	WAD	> 2	F	**+**	**+**	Yes	No	**–**
	Goat	WAD	> 2	F	**+**	**+**	Yes	Yes	PX445917
	Sheep	WAD	> 2	F	**+**	**+**	Yes	Yes	PX445918

F: Female; M: Male; WAD: West Africa dwarf; RT-cPCR: Reverse transcription conventional polymerase chain reaction (RT-qPCR): Real-time reverse transcription-polymerase chain reaction.

Among the 17 RT-cPCR positive samples, 11 (64.7%) were from goats and 6 (35.3%) from sheep. A majority (82.4%) were females, and over half (52.9%) were older than two years. All RT-cPCR-positive samples belonged to the WAD breed. The Poro and Bagoué regions each accounted for 41.2% of RT-cPCR confirmed cases, while Tchologo accounted for 17.6% (Table 1). The ten nucleotide sequences were distributed across the three regions: four from Poro, four from Bagoué, and two from Tchologo. Seven of these sequences were derived from goat samples, while only three were obtained from sheep (Table 1).

### 3.2. Phylogenetic tree

**Figure 3.**
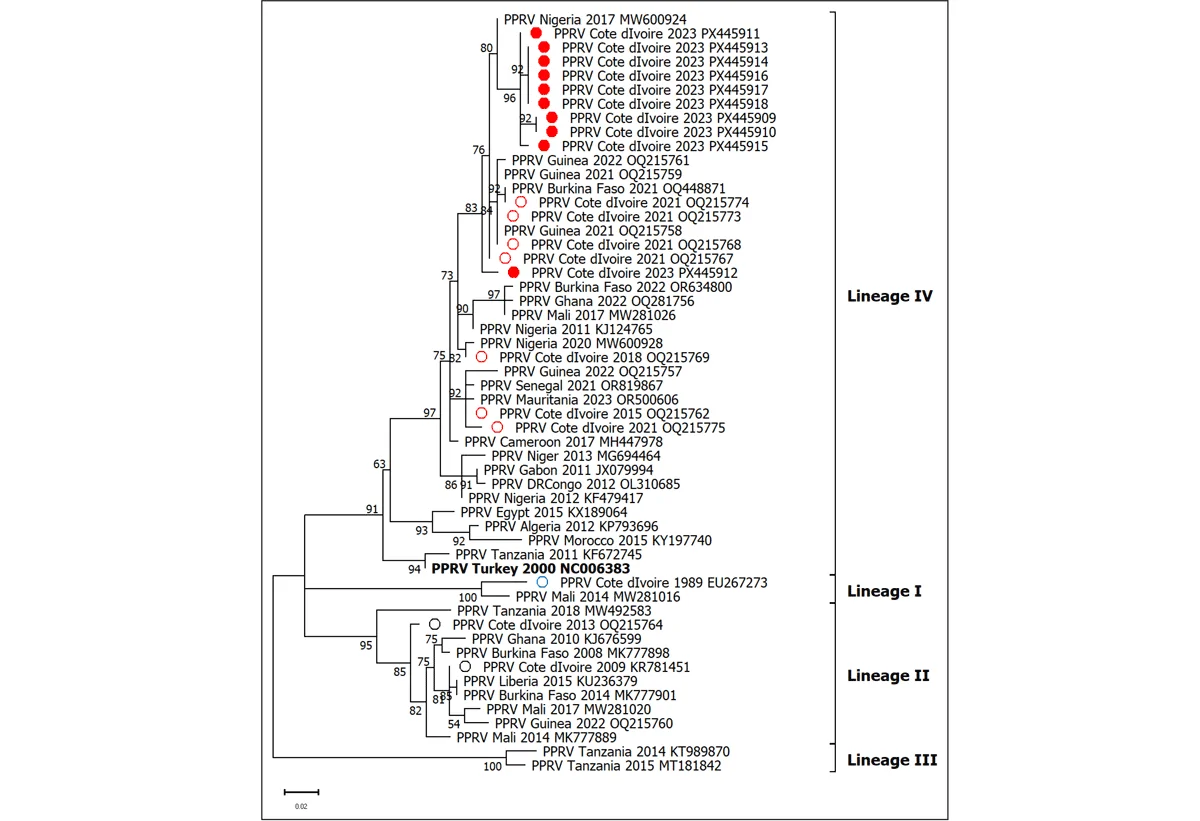
Phylogenetic tree illustrating the lineages of peste des petits ruminants virus (PPRV) based on a 228-base pair (bp) partial sequence of the *N* gene amplified from field samples. The tree includes strains from Africa and was reconstructed using the Maximum Likelihood (ML) method implemented in PhyML, employing the p-distance model. Bootstrap analysis was performed with 100 replicates, and values below 50% were excluded for clarity. Sequences generated in this study are indicated by solid red circles; previously reported lineage IV sequences from the Ivory Coast are shown as open red circles. Open black circles represent lineage II, open blue circles represent lineage I, and the reference *N* gene sequence is displayed in bold font. Only 228 bp were used for phylogenetic reconstruction because several reference sequences available in GenBank covered only this conserved region, allowing for consistent alignment and reliable comparative analysis.

The 10 sequences obtained in this study were analyzed together with forty reference sequences retrieved from GenBank to reconstruct the phylogenetic tree. The analysis, based on a 228 bp fragment of the *N* gene, revealed that all nucleotide sequences generated in this study clustered within lineage IV of PPRV, with 96–98% nucleotide identity to previously reported strains, particularly those from West Africa (Figure 3).

The resulting phylogenetic tree revealed a clear subdivision into two distinct clusters within lineage IV. The first cluster comprised a single sequence isolated from the Korhogo department (Poro region), while the second cluster grouped sequences from multiple locations, including Sinématiali (Poro region), Ferkessédougou (Tchologo region), and Boundiali and Tengréla (Bagoué region).

## 4. Discussion

With an estimated population of 4,070,402 goats and 2,531,492 sheep in 2022 [19], small ruminants play a vital socio-economic role in the Ivory Coast. They serve as an important source of income, nutrition, and livelihood, particularly for rural households. Livestock farming is practiced nationwide, with a higher concentration in the central and northern regions. In the Savannah district, livestock production is predominantly extensive, based on a traditional husbandry system that generally lacks veterinary supervision and systematic health monitoring [26].

Peste des petits ruminants was first officially reported in the Ivory Coast in 1942 [20] and continues to circulate endemically. Despite its significant economic and health impact, available epidemiological and molecular data remain limited. Apart from the studies by Couacy-Hymann et al. [15], which identified lineages II and IV, and by Gragnon et al. [21], which confirmed the virus circulation in the Korhogo department, little information exists, particularly in the Savannah District. The present study, therefore, provides new and essential insights into the molecular epidemiology of PPRV in this strategic region, which is critical for livestock production, transhumance, and the importation of live animals from Burkina Faso and Mali.

Viral RNA was initially detected using real-time RT-qPCR targeting the *N* gene of PPRV, which was used as a screening tool. Of the 25 RT-qPCR-positive samples, 17 were subsequently confirmed and successfully amplified by RT-cPCR, confirming the presence of viral RNA. Sequencing and phylogenetic analyses were then performed on a subset of ten RT-cPCR-positive samples that yielded amplicons of sufficient quality for reliable subsequent analysis. The reduction in the number of samples between screening, confirmation, and sequencing may be attributed to low viral RNA concentrations (high Ct values), RNA degradation during collection, transport, or storage, and/or insufficient amplification quality for sequencing. This stepwise approach, RT-qPCR for screening, RT-cPCR for confirmation, and sequencing for molecular characterization, ensured methodological accuracy and robustness. These findings confirm active PPRV circulation across the Poro, Tchologo, and Bagoué regions and are consistent with the previously reported seroprevalence of 36.26% [21]. This epidemiological situation may be further exacerbated by cross-border livestock movements from endemic neighboring countries such as Burkina Faso and Mali, where average PPR prevalence reaches 35.75% [40, 41].

The distribution of positive animals, 52% goats and 48% sheep, indicates significant exposure in both species, although goats are generally considered more susceptible. The predominance of the WAD breed, the high proportion of females (72%), and the large number of adults (> 2 years old, 56%) may reflect the composition of the local herd or cumulative exposure over time. Similar epidemiological patterns have been reported in Nigeria, Uganda, Algeria, and Ethiopia [42, 43, 44, 45, 46], suggesting consistent regional trends.

At the molecular level, the phylogenetic analysis revealed that all 10 sequences obtained in this study belonged to lineage IV of PPRV. This finding aligns with recent reports from multiple West African countries, including Burkina Faso [14, 15], Nigeria [47, 48], Mali and Niger [49, 50], Ghana, Guinea, and the Ivory Coast [15], as well as Senegal [51] and Mauritania [52]. Lineage IV has also been detected in Central, East, and North Africa, including the Democratic Republic of the Congo [53], Gabon (JX079994), Cameroon (MH447978), Egypt (KX189064), Morocco (KY197740), Algeria (KP189064), and Tanzania (KF672745). Cumulative evidence suggests that lineage IV is progressively replacing the historically dominant lineages I, II, and III, possibly due to enhanced viral adaptability, higher transmissibility, and the intensification of cross-border livestock movements [14, 15]. Seasonal transhumance, both official and unofficial, between the Ivory Coast and Sahelian countries facilitates the regular movement of herds in search of better pasture and water resources during the dry season [54], creating favorable conditions for virus introduction and spread.

The phylogenetic reconstruction revealed two distinct subgroups within lineage IV. The first subgroup, represented by a single sequence from Korhogo (Poro region), suggests persistent regional transmission and possible multiple viral introductions facilitated by livestock trade. The second subgroup, comprising sequences from Sinématiali, Ferkessédougou, Boundiali, and Tengréla, consisted of a genetically homogeneous cluster, indicating the localized circulation of a single dominant strain within the Savannah district.

These findings underscore the urgent need to strengthen molecular surveillance systems of PPRV in the Ivory Coast, particularly in the northern border areas. A detailed spatiotemporal characterization of circulating strains is critical to inform effective vaccination strategies and optimize disease control. The exclusive detection of lineage IV, despite the historical presence of lineages I and II, supports the hypothesis of an ongoing regional evolutionary replacement driven by a transboundary transmission dynamic.

Finally, this study underscores the value of integrating RT-qPCR, RT-cPCR, and sequencing for reliable detection and molecular surveillance of PPRV. However, further research should aim to expand the geographical and temporal scope of sampling, integrate herd management and mobility data, and conduct whole-genome sequencing to better characterize viral evolution. Such a comprehensive, cross-border approach will be essential to better understand the mechanisms of PPRV spread and achieve the global eradication goal by 2030.

## 5. Conclusions

This study, to our knowledge, represents the first molecular characterization of PPRV in the Savannah district of northern Ivory Coast. The findings confirm viral circulation across the Poro, Tchologo, and Bagoué regions, with a clear predominance of lineage IV, which is genetically related to strains previously reported in West Africa, including the Ivory Coast, Burkina Faso, Mali, Ghana, Nigeria, Guinea, Senegal, and Mauritania. The phylogenetic clustering into two distinct subgroups suggests possible multiple viral introductions, likely driven by cross-border livestock movements, highlighting their key role in the regional transmission dynamics of PPRV. The exclusive detection of lineage IV, with no evidence of historical lineages I and II, supports the hypothesis of an ongoing lineage replacement process in West Africa. These findings underscore the critical importance of sustained molecular surveillance as both an early warning system and a strategic support tool for guiding vaccination campaigns, disease control, and global PPR eradication efforts.

## Data Availability

The data presented in this study are available from the corresponding author upon reasonable request.

## List of abbreviations

µl, microliter; µM, micromolar; bp, base pair; FAO, Food and Agriculture Organization of the United Nations; ml, milliliter; min, minutes; PPR, Peste des petits ruminants; PPRV, Peste des petits ruminants virus; RNA, Ribonucleic acid; RPM, Revolutions per minute; RPV, Rinderpest virus; RSIF, Regional Scholarship and Innovation Fund; RT-cPCR, Reverse transcription conventional polymerase chain reaction; RT-qPCR, Real-time reverse transcription polymerase chain reaction; sec, seconds; VTM, viral transport media; WAD, West African dwarf; WOAH, World Organisation for Animal Health.
